# Supplementary material for: Comparison of clinical esthetic outcomes of immediately placed implants with and without immediate provisionalization in single-tooth implants of the esthetic area: A protocol for systematic review and meta-analysis
Source: PLoS One. 2021 Oct 15;16(10):e0258799. doi: 10.1371/journal.pone.0258799 (PMC8519431; doi:10.1371/journal.pone.0258799)
Supplement: Attachment — Submitted filename: PLOS ONE REVIEW.docx [file pone.0258799.s002.docx]

The topic of the manuscript has value and can attract the readers of the Plos one journal. The study plans to evaluate the oral rehabilitation with immediately placed single implants in the aesthetic region. However, the manuscript is not suitable for publication in its present form, so I am sorry to suggest a reject for its publication. Major problem with the study is that the results section is missing. However, the major revisions that are required for the resubmission of the manuscript are listed below.

**Language:** Whilst the content is clear, the standard of English in the manuscript needs to be edited.There are many errors involving spelling and grammar. The manuscript would benefit from a thorough proof-read by a native English speaker or if the authors work with a writer that has experience in writing/editing English based manuscripts for empirical peer reviewed science journals.

**Title:** In my opinion the title can be kept shorter. In my opinion: Comparison of esthetical outcomes of immediately placed single implants in the esthetic area with and without immediate provisionalization: A systematic review and meta-analysis

**Abstract:** I found abstract quite confusing and inappropriate, the abstract should be a summary of the main text. While in its present form, the authors mention how they had planned the work and it should be re-written with caution. This problem I mentioned is a major mistake.

**Introduction:**

- Introduction summarizes recent research related to topic and define the existing knowledge gap. Aim is clear highlighting what is the rationale of this work and

what is lacking in literature about the topic and what contribution can this work give to literature. However, introduction should be re-checked thoroughly for English language and fluency.

- First paragraph in my opinion, is not written well and should be written more clearly. There are many mistakes effecting the flow of the text. For example:

Line 58: Immediate implant placement, in which the implant is placed directly after the extraction, can reduce the treatment time and simplify the surgical procedure [1].

In my opinion, in immediate implantation sometimes it might be more challenging due to compromised situation of the bone (especially in the buccal aspect). But in cases when there is sufficient bone it simplifies the procedure without any doubts. It would be nice also to explain the situation from this point of view.

- Line 60: Immediate implant placement cannot change the remodeling of alveolar bone after tooth extraction and may even result in the recession of mucosa [2-4].

The authors discuss in line 60 contradictory statement with the previous sentence in line 58 but this discussion should be represented more clearly. The whole paragraph represents similar mistakes.

- Second paragraph is written more understandable. However, English should be re-checked.
- RCT: Please explain this abbreviation in line 83 (which is used for the first time in text).

**Material- Methods**

- Material- Methods should explain how the study was conducted, so it cannot be in future tense. Please apply corrections to this major problem with the text. It can be written: The study was planned as…, the work was conducted as… etc…
- I couldn’t understand the multiple parenthesis. Line 118,121
- Line 147: Primary outcome indicator~~s. The primary outcome~~ of this study will be the esthetic  outcome assessed by the objective index and patient satisfaction.
- Line 147: What is objective index? Can authors explain what they mean, it is not clear if it is PES/WES or smt additional.

**Results**

- Results section is missing

**Discussions**

- Discussions should be re-written according to the results.
